# Can Non-Translational Simplified Tasks Mimic Knee Kinematics During Gait? A Comparative Study of Tibiofemoral ICR Trajectories

**DOI:** 10.3390/biomimetics11040260

**Published:** 2026-04-09

**Authors:** Fernando Valencia, Fernando Nadal, María Prado-Novoa

**Affiliations:** 1 Clinical Biomechanics Laboratory of Andalucia (BIOCLINA), University of Málaga, 29071 Malaga, Spain; 2 Department of Mechatronics, Engineering, Faculty of Engineering in Applied Sciences, Universidad Técnica del Norte, Ibarra 100150, Ecuador

**Keywords:** tibiofemoral kinematics, instantaneous axis of rotation, instantaneous center of rotation, gait, functional tasks, motion capture

## Abstract

Understanding knee kinematics during gait is essential for the design of prostheses, orthoses, and biomimetic mechanisms. In many biomechanical analyses, tibiofemoral motion is simplified to the sagittal plane, allowing the locus of the instantaneous center of rotation (ICR) to describe joint kinematics derived from the instantaneous axis of rotation (IAR). However, it remains unclear whether ICR trajectories obtained from simplified flexion–extension tasks can represent those observed during gait. This study analyzes the sagittal-plane trajectory of the tibiofemoral ICR during gait swing, standing swing, seated swing, and squat. Motion data from 21 healthy participants were captured using videogrammetry, and the instantaneous axis of rotation (IAR) was computed from homogeneous transformation matrices using the Mozzi–Chasles theorem. Sagittal-plane ICR trajectories were derived and compared within subjects across tasks. Significant differences were found between gait and all other movements in both trajectory shape and spatial position. The shape metric (S), which quantifies differences in trajectory geometry, showed mean values ranging from 0.82 to 1.04 with very large effect sizes (Cohen’s *d* = 2.90 to 4.47, *p* < 0.0001). The centroid distance metric (M), which measures the overall spatial displacement between trajectories, indicated positional differences ranging from 8.15 mm to 12.37 mm between trajectories also showing very large effect sizes (Cohen’s = 1.72–3.40, *p* < 0.0001). Additionally, the mean deviation of the IAR from the sagittal plane ranged from 14° to 18° during gait, whereas smaller deviations were observed in non–weight-bearing swing movements. These results demonstrate that tibiofemoral ICR trajectories are task-dependent and that simplified flexion–extension tasks do not fully reproduce the knee kinematics observed during gait. Consequently, the use of gait-derived ICR trajectories, together with their variability, provides a more suitable basis for the design and optimization of polycentric mechanisms, enabling the development of devices that more closely replicate real biomechanics and are potentially better adapted to the user.

## 1. Introduction

The kinematics of the healthy human knee have been widely studied, particularly in relation to designing mechanisms for prostheses, orthoses, and exoskeletons that emulate its natural motion. Tibiofemoral flexion–extension is known to be accompanied by rotation about the other two anatomical axes: varus–valgus and internal–external rotation. Moreover, the relative motion between the tibia and femur is not a pure rotation but a combination of rotation and sliding/translation, so the tibiofemoral joint has six degrees of freedom, requiring six independent coordinates to fully describe its motion [1].

Knowledge of the locus of the relative instantaneous axis of rotation (IAR) between the tibia and the femur during flexion–extension is an important yet underexplored alternative for describing tibiofemoral motion, despite its utility in the design of biomimetic mechanisms [2,3,4,5,6,7]. To date, no studies have been found that describe the fixed and moving axoids of the joint during gait, that is, the ruled surfaces generated by the locus of the IARS with respect to a fixed or a moving reference frame.

Several studies have captured gait kinematics using stereo-radiography or fluoroscopy, tools that accurately measure femur–tibia relative position and eliminate soft-tissue artifacts. However, these technologies have limitations; they restrict the participant’s movement to a small volume-hindering natural gait and often require reduced movement speeds for technical reasons, which can affect gait kinematics [8,9,10]. Moreover, it has been reported that gait analysis should be conducted in a reproducible, quiet, well-lit environment with non-restrictive clothing, as walking speed is known to influence gait patterns [11,12,13,14].

Knee kinematics plays a fundamental role in the design of prosthetic and orthotic devices, as it directly influences joint alignment, stability, and functional performance [6,15,16]. In particular, the knee joint does not behave as a simple hinge with a fixed axis but rather exhibits a complex motion combining rolling, sliding and rotation, which results in a continuously changing ICR. Accurately capturing these kinematics characteristics is essential for achieving proper load transmission, improving gait efficiency, and reducing compensatory movements. Consequently, biomimetics design approaches seek to replicate these features to enhance the functional performance and comfort of assistive devices.

Some researchers have used dynamic X-ray imaging to record a full stride cycle during normal gait at a comfortable cadence. However, these studies have limitations stemming from the difficulty of reproducing anatomical reference frames and from the fact that only a single trial per participant was acquired to minimize radiation exposure, which hampers the assessment of within-subject variability [13,14].

Marker-based stereophotogrammetry is the most widely used technology in biomechanics laboratories to analyze human gait [17]. Although the residual calibration error of these systems can be on the order of 0.2 mm, soft-tissue artifacts (STA) can introduce errors, typically in the range of several millimeters and even exceeding 20 mm depending on the anatomical region [18,19,20]. Some authors have fixed optical markers directly to the tibia and femur to minimize this error [20,21]. However, this technique is invasive and may alter gait patterns due to pain or impingement caused by the pins [22].

In contrast, imaging-based techniques such as fluoroscopy provide higher accuracy by directly tracking bone motion; however, they are limited by restricted fields of view, radiation exposure, and experimental constraints, which hinder the acquisition of complete gait cycles under natural conditions. As a result, many studies rely on simplified movements without subject displacement, such as seated or standing swing, to approximate knee kinematics. However, it remains unclear whether these tasks adequately represent the functional kinematics of gait, particularly in terms of the trajectory of the ICR. This limitation reduces the reliability of biomechanical models used in the design of prosthetic and orthotic devices.

Inertial measurement units (IMUs) are also being used with increasing frequency, particularly for out-of-lab clinical applications. However, these sensors are susceptible to soft-tissue artifacts and vibrations, which degrade measurement accuracy. The most common methods for interpreting motion include Euler angles and the displacement of contact points on the femoral condyles [10,23,24,25,26,27]. Mean values have been reported for tibiofemoral internal–external rotation, varus–valgus rotation, and contact-point displacement during gait [10,17]. However, knee kinematics vary with task loading condition and muscle actions, which highlights the need for in vivo measurements of knee kinematics during level walking to inform the design of biomimetics mechanisms [12,17,23].

Knee motion has been modeled using sequences of rotations, yet the anatomical frames used to describe tibial and femoral motion are not consistent across studies. Reported rotation values vary with the chosen rotation order and with whether rotations are computed extrinsically or intrinsically. The Grood–Suntay convention has been widely employed to describe tibiofemoral motion; however, the definition of anatomical coordinate systems remains inconsistent [1].

Several methodologies have been used to describe the sagittal-plane locus of the tibial ICR relative to the femur, but many do not replicate natural gait conditions, which can introduce substantial errors in estimating the ICR trajectory, since the axis location is not fixed during [28,29,30]. More recently, videogrammetric recordings during natural gait have been employed, overcoming many prior limitations, albeit with simplifications such as omitting varus–valgus and internal–external rotations [5].

The objective of this study is to analyze the ICR of the tibiofemoral joint in the sagittal plane during different functional tasks and to determine whether the ICR trajectory obtained from simplified knee flexion–extension movements can accurately represent the trajectory observed during gait. Specifically, the ICR loci derived from gait swing are compared with those obtained from standing swing, seated swing, and squat movements. These comparisons are performed by evaluating differences in trajectory shape and global spatial position in order to determine whether these simplified experimental tasks can serve as reliable surrogates for gait in the characterization of knee joint kinematics. The simplified movements considered in this study do not involve spatial displacement of the subject. This characteristic makes them compatible with imaging-based measurement techniques that require the participant to remain within a fixed capture volume, such as conventional radiography, biplanar X-ray imaging systems, or fluoroscopy. Additionally, since the analysis of the ICR locus inherently simplifies the tibiofemoral joint motion to a planar movement constrained to the sagittal plane, this study also evaluates whether the validity of this simplification in gait is comparable to its validity when applied to the other movements analyzed.

The main contributions of this work are the development of a videogrammetry-based methodology to compute tibiofemoral ICR trajectories during gait, a within-subject quantitative comparison between gait and three commonly used simplified functional tasks (standing swing, seated swing, and squat) based on metrics that quantify differences in trajectory shape and spatial position and experimental evidence showing that simplified flexion–extension tasks do not accurately reproduce the tibiofemoral ICR trajectory observed during gait.

## 2. Materials and Methods

### 2.1. Participants

After IRB approval (CEUMA, Ethics Committee of the University of Málaga NR 132-2022-H), 21 healthy participants (14 male and 7 female, 27.9 ± 10.31 years old, 66.7 ± 10.87 kg in weight, 1.70 ± 0.07 m in height) were recruited for the study. All participants had no history of lower limb injuries or surgeries, no known musculoskeletal disorders, and exhibited a normal gait pattern during the initial qualitative assessment.

### 2.2. Instrumentation

The movement of lower limbs of participants was monitored in the BIOCLINA laboratory by six MX-T10 infrared cameras, equipped with a Vicon Vegas-I CMOS sensor offering a resolution of 1120 × 896. The cameras recorded at 100 Hz the positions of infrared reflective markers attached to the participants’ skin using the VICON motion capture system (Oxford Metrics ^®^, Oxford, UK). After capture, marker positions for each trial were filtered using a bidirectional 15 Hz Butterworth filter.

**Figure 1:**
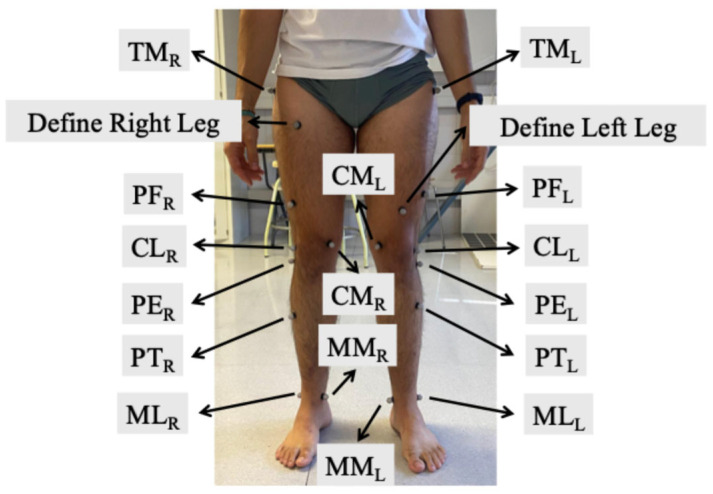
Strategic placement of reflective markers on each subject.

A set of eighteen markers, eight per leg plus two additional markers to help differentiate between the right and left leg, were defined for monitoring the relative position between tibia and femur. Marker placement was selected based on the following criteria: proximity to bony landmarks to minimize soft tissue artifact, maximize visibility throughout the trials, and avoid positions likely to experience friction during the tests. Marker placement is illustrated in Figure 1 and detailed below.

• ${TM}_{R}$, ${TM}_{L}$: center of the lateral surface of the greater trochanter of the right and left femora, respectively.
• ${CL}_{R}$, ${CL}_{L}$: center of the lateral surface of the right and left lateral femoral condyles, respectively.
• ${CM}_{R}$, ${CM}_{L}$: center of the medial surface of the right and left medial femoral condyles, respectively.
• ${PF}_{R}$, ${PF}_{L}$: on the lateral surface within the same frontal plane as respective CL and CM markers, avoiding collinearity of the three markers, and approximately in the distal third of the thigh, below the outstretched hand position so that the marker is not hidden during the trial.
• ${ML}_{R}$, ${ML}_{L}$: center of the lateral surface of the right and left lateral malleolus, respectively.
• ${MM}_{R}$, ${MM}_{L}$: center of the medial surface of the right and left medial malleolus, respectively.
• ${PT}_{R}$, ${PT}_{L}$: on the lateral surface within the same frontal plane as respective markers ML and MM, avoiding collinearity of the three markers, and approximately in the distal third of the tibia.
• ${PE}_{R}$, ${PE}_{L}$: center of the lateral surface of the head of right and left fibula, respectively.
• L, R: a reference marker was placed on the proximal third of the right thigh and the distal third of the left thigh to help in leg identification.

### 2.3. Kinematic Model

The kinematic study used a 4-link model with links associated with the following bone elements: right and left tibia; right and left femur. No kinematics constraints were established between links. Reference frames based on the markers set in Section 2.2. were established for each segment, and their position and orientation throughout the trial were computed to analyze the relative kinematics of the tibio-femoral joints.

Medial markers are prone to occlusion during trials and are susceptible to detachment from contact with the contralateral limb. While they are key for referring tibio-femoral relative motion to anatomical axis, two right-handed orthonormal reference frames were defined for each segment:

• Four non-anatomical reference frames, $\left\{f_{s}\right\}$, $\left\{t_{s}\right\}$, for the femora and tibiae, respectively, with the subscript S being L or R to refer to the left or right leg. The definition of these reference systems will not require the medial markers.
• Four anatomical reference frames, $\left\{{Af}_{s}\right\}$, $\left\{{At}_{s}\right\}$, for femoral and tibiae, the subscripts S being L or R. Calculating their position would require knowing the position of the medial markers.

The relative position between both reference systems for the same link is constant and can be defined by a homogeneous transformation matrix. All four transformation matrices can be computed from a recorded frame of an initial static trial, with the subject having the complete set of markers defined in Section 2.2. The medial markers can be removed for subsequent trials involving the movements described in Section 2.3. and the position of the anatomical reference systems can be recovered based on the position of the non-anatomical reference systems using the transformation matrices.

**Figure 2:**
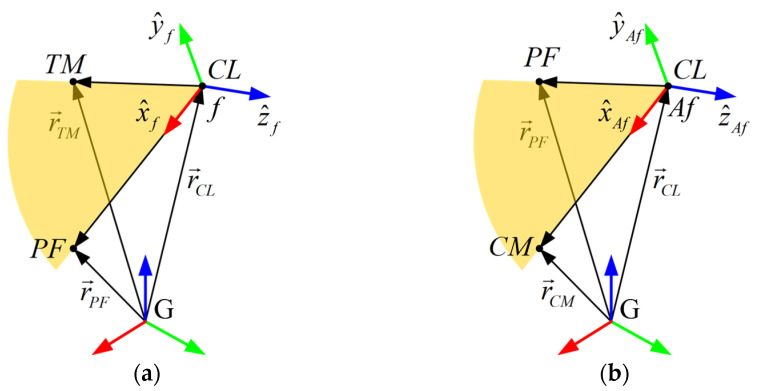
Determination of reference systems attached to the femur based on the global positions of markers CL, PF, TM, and CM. (**a**) Femur reference system {f} defined by CL, PF, and TM; (**b**) anatomical system {Af} defined by CL, PF, and C.

The orthonormal right-handed non-anatomical reference system for each femur $\left\{f_{s}\right\}$ is based on the positions of markers ${TM}_{s}$, ${CL}_{s}$, and ${PF}_{s}$, which must be computed for every studied frame. The origin is set to marker ${CL}_{s}$, whose position for the i-th frame is:

(1) $${\vec{O}}_{f}^{i}={\vec{r}}_{CL}^{i}$$

where ${\vec{r}}_{M}^{i}$ denotes the 3D vector position of marker M at the frame i-th expressed in the global reference system {G}. Unit vectors of the x, y and z axes of each femoral non-anatomic reference system (Figure 2a) at frame i-th are computed as:

(2) $${\hat{x}}_{f_{S}}^{i}=\frac{{\vec{r}}_{{PF}_{S}}^{i}-{\vec{r}}_{{CL}_{S}}^{i}}{\left|{\vec{r}}_{{PF}_{S}}^{i}-{\vec{r}}_{{CL}_{S}}^{i}\right|}$$

(3) y^fSi=r→TMSi−r→CLSi^x^fSir→TMSi−r→CLSi^x^fSi

(4) z^fSi=x^fSi^y^fSi

TRS2iRS1 is the homogeneous transformation matrix from a generic reference system RS2 to the reference system RS1 in the frame i-th. Therefore, each homogeneous transformation matrix positioning system $\left\{f_{s}\right\}$ relative to the global reference system {G} in the frame i-th is:

(5) TfSiG=x^fSiTy^fSiTz^fSiTO→fSiT0001

The femoral anatomic reference systems $\left\{{Af}_{s}\right\}$, also orthonormal and right-handed, will be computed for a selected frame 0-th of a static trial, where the full set of 18 markers must be visible. The origin is set to that of the corresponding reference system $\left\{f_{s}\right\}$ in frame 0-th, and the axes are positioned by the unit vectors (Figure 2b):

(6) $${\hat{x}}_{Af_{S}}^{0}=\frac{{\vec{r}}_{{CM}_{S}}^{0}-{\vec{r}}_{{CL}_{S}}^{0}}{\left|{\vec{r}}_{{CM}_{S}}^{0}-{\vec{r}}_{{CL}_{S}}^{0}\right|}$$

(7) y^AfS0=r→PFS0−r→CLS0^x^AfS0r→PFS0−r→CLS0^x^AfS0

(8) z^AfS0=x^AfS0^y^AfS0

Thus ${\hat{x}}_{Af_{S}}^{0}$ positions the medial–lateral axis of the femur, positive in the medial direction; ${\hat{y}}_{Af_{S}}^{0}$, the anteroposterior axis, positive in the anterior direction for the right leg and in the posterior direction for the left leg; and ${\hat{z}}_{Af_{S}}^{0}$, the craniocaudal axis, positive in the caudal direction. The homogeneous transformation matrices that position the anatomical systems of the femur $\left\{{Af}_{s}\right\}$ with respect to the global reference system {G} at the frame 0-th are:

(9) TAfS0G=x^AfS0Ty^AfS0Tz^AfS0TO→fS0T0001

The homogeneous transformation matrix between the femur reference system $\left\{f_{s}\right\}$ and the anatomical femur system $\left\{{Af}_{s}\right\}$, assuming femoral as rigid bodies, is determined by the constant matrix:

(10) TfSAfS=TAfS0G−1·TfS0G

Therefore, anatomical femoral reference systems $\left\{{Af}_{s}\right\}$ for the i-th frame can be determined from the position of the non-anatomical reference system $\left\{f_{s}\right\}$ with no need of the medial markers as:

(11) TAfSiG=TfSiG·TfSAfS−1

Reference systems for the tibiae are defined analogously. The local reference systems $\left\{t_{s}\right\}$ are defined from a subset of markers excluding the medial markers. The origin in the frame i-th is:

(12) $${\vec{O}}_{t_{S}}^{i}={\vec{r}}_{{ML}_{S}}^{i}$$

And the unit vectors of the x, y and z axes are given by:

(13) $${\hat{x}}_{t_{S}}^{i}=\frac{{\vec{r}}_{{PT}_{S}}^{i}-{\vec{r}}_{{ML}_{S}}^{i}}{\left|{\vec{r}}_{{PT}_{S}}^{i}-{\vec{r}}_{{ML}_{S}}^{i}\right|}$$

(14) y^tSi=r→PESi−r→MLSi^x^tSir→PESi−r→MLSi^x^tSi

(15) z^tSi=x^tSi^y^tSi

The homogeneous transformation matrix relating the local tibia systems to the global system is constructed for the i-th frame according to:

(16) TtSiG=x^tSiTy^tSiTz^tSiTO→tSiT0001

The anatomical reference systems of the tibia $\left\{{At}_{s}\right\}$ are computed just for frame 0-th of the static trial. The origins are set to ${\vec{O}}_{t_{S}}^{0}$ and the position of their axes will be determined by the unit vectors described as follows:

(17) $${\hat{x}}_{At_{S}}^{0}=\frac{{\vec{r}}_{{MM}_{S}}^{0}-{\vec{r}}_{{ML}_{S}}^{0}}{\left|{\vec{r}}_{{MM}_{S}}^{0}-{\vec{r}}_{{ML}_{S}}^{0}\right|}$$

(18) y^AtS0=r→PTS0−r→MLS0^x^AtS0r→PTS0−r→MLS0^x^AtS0

(19) z^AtS0=x^AtS0^y^AtS0

Thus, ${\hat{x}}_{At_{S}}^{0}$ are the medio-lateral axes, positive in the medial direction; ${\hat{y}}_{At_{S}}^{0}$ the antero-posterior axis, positive in the anterior direction for the right leg and in the posterior direction for the left leg; and ${\hat{z}}_{At_{S}}^{0}$ is the cranio-caudal axis, positive in the caudal direction.

The homogeneous transformation matrix between the anatomical tibia systems $\left\{{At}_{s}\right\}$ and the global system {G} is defined as:

(20) TAtS0G=x^AtS0Ty^AtS0Tz^AtS0TO→tS0T0001

The relationship between the two corresponding tibial reference systems $\left\{t_{s}\right\}$ and $\left\{{At}_{s}\right\}$ is constant for all frames.

(21) TtSAtS=TAtSoG−1·TtSoG

As previously discussed for the femur, once TtSAtS has been calculated at frame 0-th, the position of the anatomical reference systems of the tibiae, $\left\{{At}_{s}\right\}$, can be computed from the local systems, {$t_{s}\}$, at the i-th frame as:

(22) TGAtSi=TGtSi·TtSAtS−1

The relative position in the i-th frame of the tibial anatomical reference system with respect to the femoral anatomical reference system in the i-th frame is given by the matrix:

(23) TAfSAtSi=TGAfSi−1·TGAtSi

#### 2.3.1. Obtain the Instantaneous Axis of Rotation (IAR)

The positions of IAR of each tibiofemoral joint in the frame i-th are obtained by analyzing the movement of the tibia with respect to the corresponding femur from the frame i-th to the following frame j = i + 1. This movement is computed with respect to the anatomical axes by (24):

(24) TAfSAtSj,i=TAfSAtSj·TAfSAtSi−1=RAfSAtSj,id→AfSAtSj,i01

**Figure 3:**
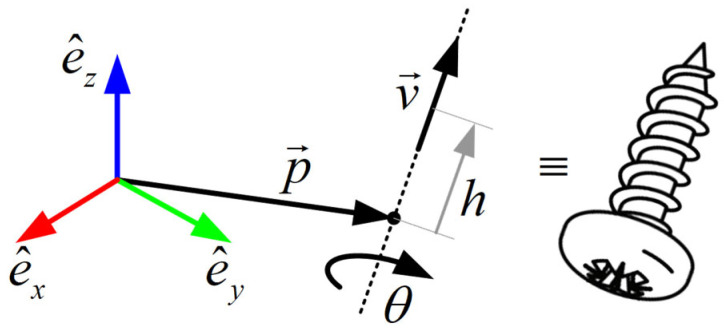
General scheme with the parameters that determine a helical motion given by a displacement h in the direction of the IAR defined by the orientation vector $\vec{v}$, and the position $\vec{p}$, of a point on the axis.

The Chasles–Mozzi theorem states that every homogeneous transformation involving a rotation and a translation can be represented by a helical motion. The transformation matrix TAfSAtSj,i involving the relative rotation of the tibia with respect to the femur RAfSAtSj,i and the relative translation d→AfSAtSj,i can be represented by the helical motion HAfSAtSj,i=v^AfSAtSj,i,w^AfSAtSj,i, as illustrated in Figure 3. Using a twist that is able to represent a combined rotational and translational transformation with a single exponential (25):

(25) TAfSAtSj,i=eHAfSAtSj,i,

where w^AfSAtSj,i=wx,wy,wz is the unit-vector representation of the skew-symmetric matrix WAtSj,iAfS defined in Equation (26).

(26) WAtSj,iAfS=0−wzwywz0−wx−wywx0,

This matrix is related to the rotational component RAfSAtSj,i as in Equation (27):

(27) RAfSAtSj,i=eWAtSj,iAfSθAtSj,iAfS,

so that the angle θAfSAtSj,i rotated by the tibia with respect to the femur can be obtained as (28), where trRAfSAtSj,i represents the trace of the matrix RAfSAtSj,i.

(28) θAfSAtSj,i=cos−1trRAfSAtSj,i−12,

The skew-symmetric matrix WAtSj,iAfS is obtained using Equation (29):

(29) WAtSj,iAfS=12sinθAtSj,iAfSRAfSAtSj,i−RAfSAtSj,iT,

The unit vector v^AfSAtSj,i defining the direction of the rotation axis, that is, the IAR of the solid, is given by (30), where the inverse of the matrix UAfSAtSj,i has the expression given in (31).

(30) v^AfSAtSj,i=UAfSAtSj,i−1·d→AfSAtSj,i,

(31) UAfSAtSj,i−1=1θAfSAtSj,iI−12WAfSAtSj,i+1θAfSAtSj,i−12cosθAfSAtSj,i2WAfSAtSj,i2,

The rotation axis is completely defined once the coordinates of a point p→AfSAtSj,i through which it passes are obtained using (32).

(32) p→AfSAtSj,i=WAfSAtSj,iv^AfSAtSj,i=w^AfSAtSj,i^v^AfSAtSj,i,

As the initial matrix TAtSj,iAfS is based on the femoral anatomical system, $\{{Af}_{S}\},$ the position of the IAR calculated in Equations (30) and (32) is relative to this system.

The knee joint flexion angle in the sagittal plane αAtSiAfS at frame i is calculated from the elements ${\left(3,2\right)}^{th}$ and ${\left(3,3\right)}^{th}$ of the rotation matrix RAtSiAfS, which is included in the transformation matrix (23). Therefore, it is necessary to consider Euler’s Equation (33) to calculate the angle given by Equation (34).

(33) RAtSiAfS=RxαAtSiAfSRyβRz(γ),

(34) αAtSiAfS=tan−1RAfS3,2AtSiRAfS3,3AtSi,

#### 2.3.2. Obtain the Instantaneous Centre of Rotation (ICR)

The IAR of the relative tibiofemoral movement intersects the sagittal plane at the ICR, whose position expressed in the femoral anatomical reference system is given for each leg by Equation (35):

(35) r→AfSICRi=p→AfSAtSj,i−v^AfSAtSj,ip→AfSAtSj,i−O→AfS·x^AfSiv^AfSAtSj,i·x^AfSi,

where O→AfS is the position of the origin of the reference system and ${\hat{x}}_{Af_{S}}^{i}$ the vector normal to the femoral sagittal planes expressed in the femoral reference systems; therefore, they become (0, 0, 0) and (1, 0, 0), respectively, for both legs.

The angle formed by the IAR with respect to the sagittal plane was calculated to evaluate the degree of simplification assumed when modeling the knee joint as a planar articulation. This angle is defined as shown in Equation (36):

(36) ϑi=cos−1r→AfSICRi·v^AfSAtSj,ir→AfSICRiv^AfSAtSj,i,

### 2.4. Testing Procedures

Once the calibration of the videogrammetric system was completed, the full set of markers described in Section 2.2. was attached to the subject, and she or he was instructed to perform the following tasks:

• Stage 1. Subject calibration: stand in static position; two static trials of approximately 1s were captured. Subsequently, the four medial markers were removed.
• Stage 2: Motion capture:
  ○ Stage 2.1: To walk on a treadmill with controlled speed at 1.44 m/s for at least 10 min. The first 3 min were not recorded to discard the period of adaptation of the volunteer to the natural gait.
  ○ Stage 2.2: The subject is trained to perform non-weight-bearing knee flexion–extension between 0 and 90º while standing on the contralateral leg (standing swing). Once trained, the volunteer completes at least 10 continuous flexion–extension cycles with each leg.
  ○ Stage 2.3: As in step 3 but with the subject seated with legs dangling (seated swing).
  ○ Stage 2.4: From an upright position, the subject performs squats, flexing to his or her maximum capacity without lifting the soles of the feet off the ground for at least 10 continuous repetitions.

### 2.5. Post-Processing

Marker positions for each trial were exported in the global {G}. All recorded trials were subdivided into containing only one movement direction, knee extension or knee flexion, and each section was analyzed independently.

For each movement section, the position of IAR was calculated at each frame using Equations (30) and (32). Likewise, the ICR positions were determined using Equation (35). The knee flexion angle was also calculated for every frame using Equation (28) and stored as a function of the knee flexion angle. Similarly, the deviation between the IAR and the sagittal plane was calculated using Equation (36).

#### Representative ICR Trajectory

The sequence of sampled points in the sagittal plane defines a curve that represents the ICR trajectory for Stages 2.1–2.4, obtained from Equation (35). For each trial, movement, and limb, 10 cycles are recorded and, from these, the representative ICR trajectories $\left\{{\left({\vec{r}}_{ICR}^{i}\right)}_{R}\right\}$ are constructed, where r→ICRi=r→ICRAfs(αAfSAtSi) and i is the index that links to the corresponding flexion angle given by Equation (34), which are defined in the femoral anatomical reference system $\left\{{Af}_{s}\right\}$.

The knee flexion-angle range is identified across the 10 trajectories obtained per trial. Let $t\in \left[1\ldots 10\right]$ denote the trajectory index, corresponding to the 10 gait cycles recorded for each movement type (extension or flexion) and limb and let $\alpha_{t}^{i}$ be the knee flexion angle associated with the i−th sample of the t−th trajectory. The analysis is restricted to the common angular range defined in Equation (37):

(37) $$\phi =\left[\alpha_{min},\alpha_{max}\right],$$

where $\alpha_{min}$ and $\alpha_{max}$ represent the minimum and maximum angular values of this common range, respectively. They are obtained from the 10 trajectories as:

(38) $$\alpha_{min}=\underset{t}{\max}\left\{\underset{i}{\min}\left(\alpha_{t}^{i}\right)\right\},\;\mathrm{and}$$

(39) $$\alpha_{max}=\underset{t}{\min}\left\{\underset{i}{\max}\left(\alpha_{t}^{i}\right)\right\},$$

• The common range was discretized using a uniform resolution of $0.1^{{}^{\circ}}$. For each trajectory, linear interpolation of the data pairs was performed over this resolution, ensuring point-to-point correspondence across the different cycles.
• The same procedure was applied to calculate the deviation between the IAR and the sagittal plane, as obtained from Equation (36).
• Finally, for each flexion angle, the point of the representative trajectory is obtained by averaging the 10 points from the curves for each of the cycles.

### 2.6. Within-Subject Comparison of ICR Trajectories

To analyze whether the knee ICR trajectory computed during gait is mimicked by the other non-translational movements described in Section 2.4., the following comparisons were performed:

1. Gait swing–extension versus standing swing–extension;
2. Gait swing–flexion versus standing swing–flexion;
3. Gait swing–extension versus seated swing–extension;
4. Gait swing–flexion versus seated swing–flexion;
5. Gait swing–extension versus squat–extension;
6. Gait swing–flexion versus squat–flexion.

In the following paragraphs, for the comparisons defined above, trajectory A represents gait (Stage 2.1), while trajectories B, C, and D correspond to standing swing (Stage 2.2), seated swing (Stage 2.3), and squat (Stage 2.4), respectively. These movements are generically denoted by the notation mov, which represents any of the movements B, C, or D when compared with gait. The direction of the knee flexion–extension motion is indicated by d, where d = E denotes extension and d = F denotes flexion.

The following calculations are performed for each subject to compute two metrics used to compare the shape and position of the tibiofemoral ICR trajectory during gait with those obtained from the other non-translational movements:

• First, representative trajectories are sampled over a common flexion range for extension or flexion defined for each pair of curves $\phi_{A,mov}$, using the discretization defined in Section Representative ICR Trajectory. Let $\alpha^{i}$ denote the i−th flexion angle in this range, with $i=1,\ldots ,N_{C}$. The corresponding ICR positions are:
  $${\vec{r}}_{A_{d}}\left(\alpha^{i}\right)={\left({\vec{r}}_{ICR}\left(\alpha^{i}\right)\right)}_{A_{d}}$$
  $${\vec{r}}_{{mov}_{d}}\left(\alpha^{i}\right)={\left({\vec{r}}_{ICR}\left(\alpha^{i}\right)\right)}_{{mov}_{d}}$$
  where mov ∈ {B, C, D}. The common flexion range $\phi_{A,mov}$ for each pair of movements is defined as the intersection of the angular domains of both representative trajectories.
• The length $L_{A,mov}$ is obtained by accumulating the lengths of the segments between two consecutive instants across the entire common range, as defined in Equation (40), where $N_{C}$ is the size or number of elements in the sequence of values that define the range $\phi_{A,mov}$.
  (40) $$L_{A_{d},{mov}_{d}}=\sum_{i=1}^{N_{C}-1}\left\|{{\vec{r}}_{A}^{i}}_{d}-{{\vec{r}}_{mov}^{i}}_{d}\right\|$$
• The centroids of each pair of trajectories are computed according to Equation (41).
  (41) $${{\vec{C}}_{R}}_{d}=\frac{1}{N_{C}}\sum_{i=1}^{N_{C}}{{\vec{r}}_{R}^{i}}_{d}$$
• The Euclidean distance $M_{A,mov}$ between pairs of centroids is computed as expressed in Equation (42) and used as a metric to quantify the positional similarity between ICR trajectories.
  (42) $$M_{A_{d},{mov}_{d}}=\left\|{{\vec{C}}_{A}}_{d}-{{\vec{C}}_{mov}}_{d}\right\|$$
• To normalize the trajectories, they are first translated so that their centroids are located at the origin. Then, the coordinates of the points are scaled by a factor of $1/L_{A,mov}$ to obtain the normalized coordinates ${{\hat{r}}_{A}^{i}}_{d}$, and ${{\hat{r}}_{mov}^{i}}_{d}$ for each point.
• For each segment of the trajectories (i≥3), three consecutive points are considered, and the direction vectors are defined as ${\vec{v}}_{A,i-1}={\hat{r}}_{A_{d}}^{i-1}-{\hat{r}}_{A_{d}}^{i-2}$ and ${\vec{v}}_{mov,i-1}={\hat{r}}_{{mov}_{d}}^{i-1}-{\hat{r}}_{{mov}_{d}}^{i-2}$. The difference between their local directions, the angle λ between corresponding segments, is given by Equation (43).
  (43) $$\lambda_{i-1}=\tan^{-1}\left(\frac{\left\|{\vec{v}}_{mov,i-1}\times {\vec{v}}_{A,i-1}\right\|}{{\vec{v}}_{mov,i-1}\cdot {\vec{v}}_{A,i-1}}\right)$$
• The translation that aligns the direction on the local segments ${\vec{v}}_{A,i-1}$ with ${\vec{v}}_{mov,i-1}$ and the position of the points i=1 in the YZ plane is computed. This translation is applied only to ${\vec{v}}_{mov,i}$. The distance between point i of trajectory A and the same flexion angle points i of trajectory mov∈{B,C,D} are computed. Accumulating these differences over all points yields a global metric $S_{A,mov}$, defined in Equation (44), which reflects the degree of shape similarity between trajectories.
  (44) $$S_{A_{d},{mov}_{d}}=\sum_{i=3}^{N}\left\|R_{i}\left({\hat{r}}_{{mov}_{d}}^{i}-{\hat{r}}_{{mov}_{d}}^{i-1}\right)-\left({\hat{r}}_{A_{d}}^{i}-{\hat{r}}_{A_{d}}^{i-1}\right)\right\|$$

According to the procedure described above, for each of the N tested individuals, six metrics quantifying differences in ICR trajectory shape and six metrics quantifying differences in trajectory position were obtained for the six comparisons listed at the beginning of this section.

For the metric (S), trajectories were normalized by their corresponding length, resulting in dimensionless quantities. For each subject and limb, trajectory-level values were first averaged to obtain a representative value per condition. Subsequently, group-level statistics (mean ± standard deviation) were computed across these subject–limb averages.

It should be noted that the shape metric used in this study was specifically developed for this work to quantify local differences between trajectories. Therefore, it is not derived from a previously established method. However, its formulation is conceptually related to existing approaches for curve comparison based on point-wise differences and cumulative measures along discretized trajectories.

For the metric (M), the same averaging procedure was applied; however, trajectories were not normalized and, therefore, the resulting values are expressed in millimeters.

Finally, the mean deviation angle from the sagittal plane is computed over the common knee flexion range defined in Equation (36), considering the 10 ICR trajectories corresponding to each movement, limb, and subject. This quantity is denoted as ${\bar{\vartheta }}_{A_{d}}$ for gait swing and as ${\bar{\vartheta }}_{{mov}_{d}}$ for the other movements.

### 2.7. Statistical Analysis

The differences between the ICR trajectory in the sagittal plane during gait and in the other three studied movements without subject displacement are assessed by analyzing statistical differences in the two metrics defined in Section 2.6., related to ICR trajectory shape and position.

The difference in trajectory shape between gait and the compared movement is quantified by the metric $\;S_{A_{d},{mov}_{d}}$ computed for each N individuals. The statistical hypotheses are defined as:

**Hypothesis** **1.**
$H_{0}:\;\mu S=0$ *(no average difference in trajectories shapes).*

**Hypothesis** **2.**
H1: μS>0 *(a significant difference in trajectories shape exists).*

The positional difference between the ICR trajectories is quantified by the centroid distance metric $M_{A_{d},{mov}_{d}}$ computed for each N individuals. The statistical hypotheses are defined as:

**Hypothesis** **1.**
H0: μM=0 *(no average difference in centroid positions).*

**Hypothesis** **2.**
H1: μM>0 *(a significant displacement between centroids exists).*

A one-tailed formulation was adopted, since both metrics are strictly non-negative by definition.

For the data analysis, the one-sample *t*-test was used for those variables $M_{A_{d},{mov}_{d}}$ that approximately followed a normal distribution. In cases where the variables did not follow a normal distribution, the non-parametric Wilcoxon signed-rank test was applied. Additionally, to assess the normality of distribution of $M_{A_{d},{mov}_{d}}$ and $S_{A_{d},{mov}_{d}}$, the Shapiro–Wilk test was used.

Additionally, the effect size (Cohen’s d) is used to express a standardized measure of the actual magnitude of a difference, that is, how far the mean of each variable departs from a reference value (in this case, how far $M_{A_{d},{mov}_{d}}$ or $S_{A_{d},{mov}_{d}}$ are from 0) expressed in units of standard deviation.

Accordingly, the *p*-value from the *t*-test or the Wilcoxon test is used to verify whether the difference between $M_{A_{d},{mov}_{d}}$ or $S_{A_{d},{mov}_{d}}$ and 0 is statistically significant (*p* < 0.05 is commonly used to consider differences significant), and Cohen’s d, the effect size, is used to quantify the practical/experimental magnitude of that difference.

Effect size is commonly computed as defined in Equation (45):

(45) $$d=\frac{\bar{x}-\mu_{0}}{s}=\frac{\bar{x}}{s},$$

In this case the null hypothesis ($\mu_{0}$) is 0. In practice, d<0.2 is considered a very small effect size (virtually no difference), and d>0.8 is considered a large effect size (strong or relevant difference).

Additionally, it was analyzed whether the deviation from the sagittal plane of knee flexion–extension in the three movements other than gait, expressed in terms of the mean angle ${\bar{\vartheta }}_{{mov}_{d}}$, was comparable to that observed during gait, ${\bar{\vartheta }}_{A_{d}}$. This was assessed using the same six statistical comparisons previously defined between gait and the other non-translational movements within the common knee flexion range.

Given the paired nature of the data, a paired Student’s *t*-test was applied to evaluate whether the mean difference between movements was significantly different from zero. The normality of the paired differences was assessed prior to the analysis. As a non-parametric contrast, the Wilcoxon signed-rank test was additionally performed to test the null hypothesis of zero median difference. For each comparison, the test statistic and the corresponding *p*-value were reported.

## 3. Results

### 3.1. Representative ICR Trajectories

**Figure 4:**
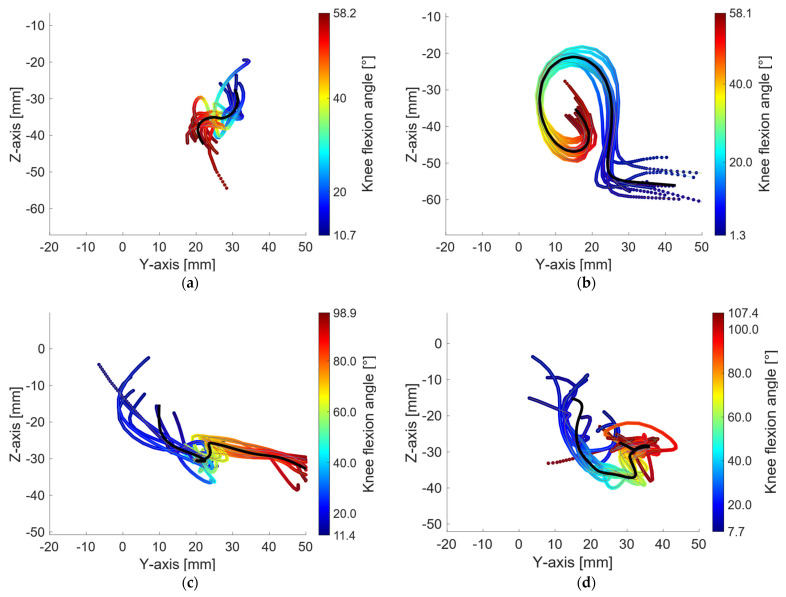

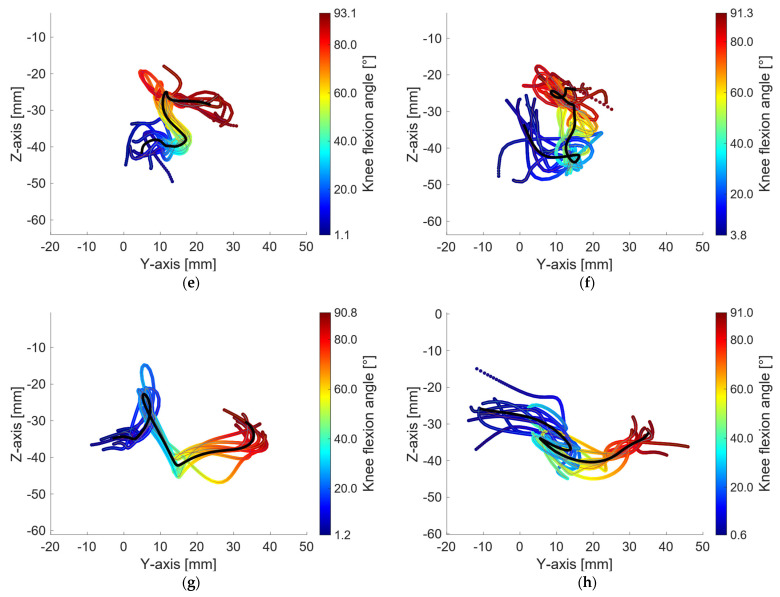
Representative ICR trajectories: (**a**) gait in extension, with a flexion-angle range from 10.7° to 58.2°; (**b**) gait in flexion, with a flexion-angle range from 1.3° to 58.1°, (**c**) swing in extension, with a flexion-angle range from 11.4° to 98.9°, (**d**) swing in flexion, with a flexion-angle range from 7.7° to 107.4, (**e**) squat in extension, with a flexion-angle range 1.1° to 93.1°, (**f**) squat in flexion, with a flexion-angle range 3.8° to 91.3°, (**g**) seated in extension, with a flexion-angle range from 1.2° to 90.8°, (**h**) seated in flexion, with a flexion-angle range from 0.6° to 91°.

Below, the 10 trajectories computed for each studied trial of each leg are shown as illustrative examples of the obtained results for subject M19LR, right leg: gait in extension (Figure 4a), gait in flexion (Figure 4b), swing in extension (Figure 4c), swing in flexion (Figure 4d), squat in extension (Figure 4e), squat in flexion (Figure 4f), seated in extension (Figure 4g), and seated in flexion (Figure 4h). From these data, the mean ICR trajectory $\left\{{\left({\vec{r}}_{ICR}^{i}\right)}_{R}\right\}$, shown as the black trajectory in Figure 4, is obtained. Trajectories are expressed as a function of the flexion angle, which is linearly interpolated with a resolution of 0.1° and over a common flexion range for all trajectories within each case, as described in Section Representative ICR Trajectory.

During the paired comparisons between movements, some trials were excluded due to missing frames that prevented full coverage of the common flexion range required. Consequently, the sample prevented full coverage of the common flexion range required for analysis. Consequently, the sample size varied across comparisons.

For the metrics $\left(S\right)$ and (M), the sample sizes were: N = 40 for gait–standing (extension), N = 41 for gait–standing (flexion), N = 39 for gait–seated (extension), N = 40 for gait–seated (flexion), N = 40 for gait–squat (extension), and N = 41 for gait–squat (flexion).

For the angular deviation between the IAR and the sagittal plane, the sample sizes were: N = 40 for gait–standing (extension), N = 40 for gait–standing (flexion), N = 38 for gait–seated (extension), N = 36 for gait–seated (flexion), N = 39 for gait–squat (extension), and N = 39 for gait–squat (flexion).

### 3.2. Differences in ICR Trajectory Shape with Respect to Gait

Following the procedure described in Section 2.6, the similarity in curve shape between the representative ICR trajectory during gait and those obtained from the other movements was evaluated separately for knee extension and knee flexion using the shape metric $S_{A_{d},{mov}_{d}}$ described in Equation (44).

Across all comparisons, the distribution of $S_{A_{d},{mov}_{d}}$ was compatible with normality (Shapiro–Wilk, *p* > 0.05); therefore, a one-sample, one-tailed *t*-test was applied to assess whether the mean difference in trajectory shape was significantly greater than zero. The results of these comparisons are summarized in Table 1, which reports the mean, standard deviation (SD), 95% confidence interval (CI) and effect sizes.

A highly significant difference was observed during gait and the other movements (*p* < 0.0001). Moreover, the effect sizes (Cohen’s d) were consistently very large, indicating substantial differences in trajectory shape between gait and the other tasks.

### 3.3. Differences in ICR Trajectory Position with Respect to Gait

Following the comparison procedure described in Section 2.6, the positional differences between ICR trajectories obtained during gait and those derived from the other movements were quantified using the centroid distance metric $M_{A_{d},{mov}_{d}}$, defined in Equation (42).

The Shapiro–Wilk test indicated a clearly non-normal distribution of the datasets (*p* < 0.05); therefore, a Wilcoxon signed-rank test was used as well as a one-sample *t*-test as a contrast. The statistical results for all comparisons are summarized in Table 2, which reports the mean values, standard deviations, confidence intervals, and effect sizes.

For all comparisons, the centroid distance metric revealed statistically significant positional differences between the ICR trajectories obtained during gait and those obtained from the other movements., The effect size (Cohen’s d) was very large in all cases, indicating that the spatial position of the ICR trajectory during gait differs substantially from that observed in the other functional tasks.

It should be noted that the reported effect sizes differ between Table 1 and Table 2 because they correspond to different metrics. Table 1 evaluates differences in trajectory shape (S), while Table 2 assesses differences in the global spatial position (M). Therefore, effect sizes are not expected to be identical, although both consistently indicate significant differences between movements.

### 3.4. Differences in IAR Deviation from the Sagittal Plane with Respect to Gait

Table 3 summarizes the mean angular deviation of the IAR from the sagittal plane for each pairwise comparison within the common knee flexion range. For each movement condition, the reported values (mean ± SD) correspond to the average deviation angle computed across the 10 ICR trajectories of each subject and limb and subsequently pooled across subjects. The sample size (N) varies slightly between comparisons due to differences in the number of valid trajectories available within the common flexion range for each movement phase.

Paired Student’s *t*-tests were used as the primary inferential analysis, with the Wilcoxon signed-rank test applied as a non-parametric contrast. Both methods yielded consistent conclusions across all comparisons.

During gait, the mean deviation ranges between 14° and 18°, confirming that knee flexion–extension during walking is not a purely planar motion, while standing swing and seated swing exhibit significantly smaller deviation angles. The statistical comparisons show significant differences between gait and these two movements in both extension and flexion phases (*p* < 0.05), indicating that swing movements performed without spatial displacement of the subject tend to produce a motion that is closer to the sagittal plane.

A different behavior is observed for the squat movement. In this case, no statistically significant differences were detected between squat and gait in either extension or flexion; however, the mean deviation angles were numerically comparable. This suggests that squatting, a weight-bearing task, may reproduce a level of three-dimensionality in knee kinematics similar to that observed during walking. Consequently, among the non-translational movements analyzed, squat may better preserve the spatial characteristics of knee motion associated with gait, whereas swing movements performed without load tend to result in a more planar representation of the joint kinematics.

## 4. Discussion

The main finding of this study is that the sagittal-plane ICR trajectory obtained during gait cannot be accurately represented by the trajectories derived from other commonly analyzed movements with no subject spatial displacement, specifically standing-swing, seated-swing, and squat, which are frequently used as experimental alternatives due to their feasibility in imaging-based techniques. Consequently, the use of these non-gait movements as surrogates for gait leads to a non-negligible deviation in the representation of knee joint kinematics in the sagittal plane. These findings indicate that ICR trajectories are task-dependent and not interchangeable across movements, highlighting a fundamental limitation in extrapolating gait kinematics from constrained experimental tasks.

In this study, the motion of both legs of 21 subjects was recorded, resulting in a dataset comprising 42 lower limbs, for the following movements: normal gait, swing with the subject seated, swing with the subject standing on the contralateral leg, and squatting. The data acquisition was performed using a non-invasive approach based on surface markers tracked by a videogrammetry system. Subsequently, the IAR of the relative motion between the femur and the tibia was determined, considering the extension and flexion phases separately. As is commonly assumed in gait analysis studies used for the design of prostheses for amputee patients, knee flexion–extension was simplified to a planar motion in the sagittal plane, and the geometric locus of the ICR was computed in this plane. For all comparisons between gait and the other analyzed tasks, the shape metric $S_{A_{d},{mov}_{d}}$ yielded positive values significantly greater than zero, with highly significant *p*-values. These results indicate that the geometric shape of the ICR trajectory during gait cannot be reproduced by the trajectories derived from the other movements analyzed. Moreover, the large effect sizes obtained in all cases confirm that these differences are not only statistically significant but also biomechanically relevant. Likewise, the centroid distance metric $M_{A_{d}{,mov}_{d}}$ yielded values significantly greater than zero, with statistically significant *p*-values. These findings demonstrate that, in addition to differences in trajectory shape, the global spatial position of the ICR trajectories also varies between gait and the other studies tasks. The magnitude of the effect sizes further supports the practical relevance of these positional differences. Overall, these results indicate that the spatial location of the ICR trajectory is task-dependent and that movements such as standing swing, seated swing, and squat do not reproduce the same global position of the trajectory observed during gait.

The consistently large effect sizes observed across all comparisons (Cohen’s d > 1.7 and up to 4.47) indicate that the differences between gait and the analyzed simplified movements are not only statistically significant but also represent substantial biomechanical discrepancies. These magnitudes suggest that the ICR trajectory during gait is fundamentally distinct in both shape and spatial location, rather thar being a variation in a common underlying pattern. Consequently, the use of simplified flexion–extension tasks as surrogates for gait may lead to systematic errors in the representation of knee joint kinematics.

Additionally, the deviation of the flexion–extension movement from the sagittal plane was analyzed. Evaluating this deviation is crucial for assessing the suitability of the common simplification that three-dimensional knee joint kinematics can be reduced to the sagittal plane. In the scientific literature, to the best of the authors’ knowledge, no studies have specifically examined whether non-translational knee movements preserve the three-dimensional characteristics of real gait kinematics. The results of this study suggest that movements performed without load, such as standing swing and seated swing, tend to simplify knee motion toward a more planar behavior, reducing the out-of-plane components of the instantaneous axis of rotation. In contrast, weight-bearing movements such as squatting exhibit deviations from the sagittal plane comparable to those observed during gait, indicating that they better preserve the three-dimensional nature of knee kinematics. These findings suggest that non-weight-bearing tasks may provide an oversimplified representation of knee motion when used as substitutes for gait analysis.

Numerous studies have analyzed knee kinematics during different movements. However, few have calculated the IAR and, to the best of our knowledge, none have compared the ICR in the sagittal plane during gait with that obtained from other movements, which are commonly used in studies other than videogrammetric approaches, such as those based on X-ray imaging or fluoroscopy.

X-ray and fluoroscopic studies do not allow easy gait analysis because the measurement field in which the subject must remain is very limited, restricting the analysis to movements such as swing, seated, standing or squat. In contrast, these techniques enable direct visualization of the bony surfaces, eliminating skin-motion artifacts and providing higher accuracy than systems based on surface markers.

Gasparutto et al. [31] present a synthesis of data derived from different experimental studies, based on averaged curves that do not represent the kinematics of a specific subject. In addition, these curves were digitized from previously published graphs, and the reconstructed trajectories rely on a single cadaveric knee geometry, without a clearly defined anatomical reference frame, which limits their practical reproducibility and their direct applicability to intra-subject analyses.

On the other hand, studies such as that in [12], based on biplanar radiography and a sample of 39 knees during walking, have demonstrated that, even in healthy knees, there is considerable inter-subject variability in tibiofemoral kinematics. In this context, the instantaneous center of rotation (ICR) trajectory obtained in the present study falls within these ranges of variation for the walking condition, reinforcing the interpretation of the ICR as a feature that depends on both the subject and the type of movement, rather than as a fixed and universal pattern.

Likewise, the study in [32] employed dual fluoroscopy to investigate six-degrees-of-freedom kinematics and condylar motion of the knee during the stance phase of treadmill walking, demonstrating that joint kinematics are highly activity-dependent and that movement patterns cannot be generalized across different tasks. However, this study does not establish an explicit anatomical reference frame, nor does it address the representation of knee kinematics in the sagittal plane as a functional simplification of the instantaneous center of rotation (ICR), an approach widely used in the design and development of medical devices. In contrast, the present study quantifies the angular deviation associated with this simplification, thereby enabling an assessment of its effect on the functional description of joint motion.

Similarly, [33] present a non-invasive dual-fluoroscopy technique for the analysis of six-degrees-of-freedom knee kinematics during treadmill walking. However, the number of subjects analyzed is limited, and the study focuses primarily on the validation of the imaging system rather than on the quantification of kinematic simplifications applicable to functional models. To date, no studies have been identified that explicitly quantify the degree of simplification of three-dimensional knee kinematics toward a sagittal-plane representation of the instantaneous center of rotation (ICR), despite its relevance for the development of external medical devices, such as subject-specific external knee prostheses, as those developed by [6].

The methodology employed in this study is entirely non-invasive, as it is based on the use of surface markers, which allows gait to be evaluated under real functional conditions. Nevertheless, this approach is subject to the well-known soft tissue artifact, which may affect the accuracy of estimating fine joint translations. Even so, several studies have reported that, during gait, global movement patterns and the main kinematic couplings are preserved, even in the presence of such artifacts [18,34].

In contrast, conventional fluoroscopic methods present significant limitations, as the restricted field of view, radiation exposure, and experimental constraints hinder the acquisition of complete gait cycles or large lower-limb movements. These limitations generally confine the analysis to highly controlled conditions, preventing the continuous and unrestricted recording of leg motion during prolonged displacements, such as those occurring during walking [35].

The methodological approach adopted in the present study allows the analysis of multiple gait cycles per participant and the exploration of intra-subject variability, providing a robust description of the ICR trajectory under real dynamic conditions.

The results presented in this study were obtained using a treadmill at a predefined constant speed. In this regard, it should be acknowledged that overground gait may lead to differences in knee kinematics, which could slightly modify the ICR trajectory. Therefore, future studies should consider analyzing gait under overground conditions and at different speeds, as both walking speed and cadence may influence the trajectory of the IAR and, consequently, its projection onto the sagittal plane ICR.

Regarding the phase coverage of the gait cycle, the present study focused primarily on the swing phase due to the greater stability of IAR estimation. During the stance phase, particularly at initial contact (heel strike), inconsistencies were observed in the data, associated with impacts and possible marker occlusions, which affect the robustness of IAR. For this reason, the analysis of the stance phase was limited in this study.

The results of this study provide direct guidance for the design and evaluation of prosthetic, orthotic, and biomimetic knee mechanisms. The observed task-dependent differences in ICR trajectories indicate that simplified flexion–extension tasks, such as seated or standing swing, should not be used to define or tune mechanism geometry for gait applications. Designs based on such conditions may fail to reproduce the functional tibiofemoral kinematics required for gait.

Instead, gait-derived ICR trajectories should serve as the primary reference for mechanism synthesis, particularly during the swing phase, where dynamic conditions are critical. Incorporating both the mean trajectory and its variability may enhance the adaptability of polycentric mechanisms across different users and movement conditions.

From a measurement perspective, capturing multiple gait cycles per subject enables the assessment of intra-subject variability, which is essential for identifying representative kinematic patterns. This supports the use of non-invasive motion capture systems as a practical tool for generating design-relevant kinematic datasets under realistic conditions.

## 5. Conclusions

The proposed methodology enables non-invasive estimation of the instantaneous axis of rotation (IAR) between the femur and tibia during flexion in vivo, characterizing normal gait kinematics in the sagittal YZ plane as the trajectory of the instantaneous center of rotation (ICR).

The ICR curves corresponding to gait-swing, when compared with standing-swing, seated-swing, and squat, differs significantly in both shape and position. Positive S and M values, together with the effect size, indicate robust and consistent differences between the curves of the compared movements, with strong experimental relevance.

The results show that the ICR trajectory differs across movements and cannot be accurately represented by knee flexion derived from tasks such as standing-swing, seated-swing, and squat. This indicates that the ICR trajectory is task-dependent and that these simplified tasks are not suitable surrogates for characterizing gait kinematics.

In addition, the angular variation associated with the simplification of three-dimensional knee joint kinematics to a sagittal-plane ICR representation was quantified for the studied movements. This analysis provides further insight into the functional implications of adopting a planar representation of knee joint kinematics under dynamic conditions.

## Figures and Tables

**Table 1:** Results for the shape metric $S_{A_{d},{mov}_{d}}$ comparing ICR trajectories during gait and the other movements for flexion and extension.

Condition	Mean	SD	IC 95%	Normality (W, *p*)	t (N)	*p* (One-Tailed)	Effect Size
Gait-swing vs. Standing-swing (EXTENSION)	0.82	0.28	(0.73, 0.92)	0.97; 0.42	18.35 (40)	<0.0001	Cohen’s d = 2.90
Gait-swing vs. Standing-swing (FLEXION)	1.04	0.30	(0.94, 1.13)	0.97; 0.37	21.77 (41)	<0.0001	Cohen’s d = 3.40
Gait-swing vs. Seated-swing (EXTENSION)	0.87	0.27	(0.78, 0.96)	0.97; 0.52	20.09 (39)	<0.0001	Cohen’s d = 3.22
Gait-swing vs. Seated-swing (Flexion)	1.04	0.23	(0.96, 1.11)	0.04; 0.03	28.24 (40)	<0.0001	Cohen’s d = 4.47
Gait-swing vs. Squat (EXTENSION)	0.96	0.30	(0.86, 1.05)	0.97; 0.30	19.87 (40)	<0.0001	Cohen’s d = 3.14
Gait-swing vs. Squat (FLEXION)	1.06	0.32	(0.96, 1.16)	0.96; 0.17	21.03 (41)	<0.0001	Cohen’s d = 3.28

**Table 2:** Results for the positional metric $M_{A_{d},{mov}_{d}}$ comparing ICR trajectories during gait and the other movements for flexion and extension.

Condition	Media [mm]	SD [mm]	IC95%	Normality (W, *p*)	Wilcoxon	*p* (One-Tailed Cola)	t (N)	*p* (aux)	Effect Size
Gait-swing vs. Standing-swing (EXTENSION)	8.84	4.90	(7.28, 10.41)	0.95; <0.0001	820.0	<0.0001	11.42 (40)	<0.0001	Cohen’s d = 1.81
Gait-swing vs. Standing-swing (FLEXION)	11.34	6.79	(9.20, 13.48)	0.85; <0.0001	861.0	<0.0001	10.70 (41)	<0.0001	Cohen’s d = 3.40
Gait-swing vs. Seated-swing (EXTENSION)	12.37	5.02	(10.70, 13.95)	0.98; <0.0001	780.0	<0.0001	15.35 (39)	<0.0001	Cohen’s d = 2.46
Gait-swing vs. Seated-swing (FLEXION)	8.15	4.26	(6.79, 9.52)	0.95; <0.0001	820.0	<0.0001	12.10 (40)	<0.0001	Cohen’s d = 1.91
Gait-swing vs. Squat (EXTENSION)	11.96	4.29	(10.59, 13.33)	0.97; <0.0001	820.0	<0.0001	17.65 (40)	<0.0001	Cohen’s d = 2.79
Gait-swing vs. Squat (FLEXION)	8.86	5.14	(7.23, 10.48)	0.93; <0.0001	861.0	<0.0001	11.03 (41)	<0.0001	Cohen’s d = 1.72

**Table 3:** Angle of deviation between the IAR and the sagittal plane.

Movements	${\bar{\vartheta }}_{A_{d}}\pm SD$	${\bar{\vartheta }}_{{mov}_{d}}\pm SD$	Wilcoxon *p*	w	Pairedt-test, t(df)
Gait-swing vs. Standing-swing (EXTENSION)	14.15°±4.28°	11.74°±3.96°	0.004	197	$t\left(39\right)=-2.9336,\;\;p=0.0055$
Gait-swing vs. Standing-swing (FLEXION)	16.88°±6.05°	12.47°±4.48°	0.0003	675	$t\left(39\right)=4.2466,\;\;p=0.0001$
Gait-swing vs. seated-swing (EXTENSION)	14.48°±4.5°	11.23°±4.9°	0.002	580	$t\left(37\right)=3.5554,\;\;p=0.0010$
Gait-swing vs. seated-swing (FLEXION)	17.05°±4.9°	8.39°±5°	0.007	504	$t\left(35\right)=2.3237,\;\;p=0.0260$
Gait-swing vs. squat (EXTENSION)	14.70°±4.17°	15.35°±11.2°	0.706	417	$t\left(38\right)=-0.4054,\;\;p=0.6874$
Gait-swing vs. squat (FLEXION)	17.7°±5.4°	17.9°±9.8°	0.647	402	$t\left(38\right)=-0.6396,\;\;p=0.5262$

These values correspond to the number of paired observations used in each statistical test (df = N − 1 for the paired *t*-test).

## Data Availability

The data presented in this study are available on request from the corresponding authors.

## Abbreviations

The following abbreviations are used in this manuscript:

ICR	Instantaneous Center of Rotation
IAR	Instantaneous Axis of Rotation
IMU	Inertial Measurement Units
